# Development of Software for the In-Depth Analysis of Protein Dynamics as Determined by MALDI Mass Spectrometry-Based Hydrogen/Deuterium Exchange

**DOI:** 10.5702/massspectrometry.S0082

**Published:** 2020-02-14

**Authors:** Tatsuya Yamamoto, Tohru Yamagaki, Honoo Satake

**Affiliations:** 1 Bioorganic Research Institute, Suntory Foundation for Life Sciences, Kyoto 619–0284, Japan

**Keywords:** HDX, MALDI, Scipas DX, MS analysis software

## Abstract

Hydrogen/deuterium exchange (HDX) coupled with pepsin digestion is useful for rapidly analyzing the kinetic properties of small amounts of protein. However, the analysis of HDX by matrix-assisted laser desorption/ionization (MALDI) mass spectrometry (MS) is time-consuming due to a lack of dedicated software. Currently available software programs mainly calculate average mass shifts, even though the isotopic distribution width contains information regarding multiple protein conformations. Moreover, HDX reaction samples are typically composed of peptides that contain various numbers of deuterium atoms, which also hinders the rapid and comprehensive analysis of protein dynamics. We report here on the development of a software program “Scipas DX” that can be used to automatically analyze the hydrogen–deuterium isotopic distribution in peaks in HDX spectra and calculate the average number of atoms exchanged, the average deuteration ratio, the abundance ratio for exchanged atoms, and their fitted spectra with a high degree of accuracy within a few minutes. Analysis of the abundance ratio for exchanged atoms of a model protein, adenylate kinase 1, using Scipas DX indicate that the local structure at residues 83–106 and 107–117 are in a slow equilibrium, suggesting that these regions adopt multiple conformations that are involved in the stability and in switching between the active and inactive forms. Furthermore, precise HDX kinetics of the average deuteration ratio both confirmed the known induced conformations of two regions (residues 46–75 and 131–165) that are responsible for ligand binding and verified the novel structural dynamics of residues 107–117 and 166–196 following ligand binding to ligand-binding pockets 1 and 2, respectively. Collectively, these results highlight the usefulness and versatility of Scipas DX in MALDI-MS HDX-based analyses of protein dynamics.

## INTRODUCTION

Protein structures have variable responses to both endogenous factors and exogenous stimuli, and these responses play essential roles in how a protein functions in the regulation of biological systems. A growing body of reports has elucidated the static tertiary structures of various proteins by X-ray crystallography and cryo-electron microscopy. However, these static structures provide only limited information regarding the molecular mode of the actions and dynamics of proteins under physiological conditions. Hydrogen/deuterium exchange (HDX) is a powerful method for observing protein structure dynamics in solution. Exchangeable hydrogen atoms on proteins are replaced by deuterium in a D_2_O solution under various conditions and the resulting data provide insights into protein dynamics and solvent accessibility. Mass spectrometry (MS) is one of the most popular techniques for detecting the results of HDX reactions.^1,2)^ HDX-MS typically provides the average change in mass resulting from the replacement of exchangeable amide hydrogen atoms with deuterium atoms on the main chain of the protein because the other exchangeable hydrogen atoms are fully exchanged when the reaction is quenched at an acidic pH.^1,3)^ Consequently, HDX-MS techniques have widely been employed for the dynamic analysis of both whole proteins and specific sites of interest (*e.g.*, protease-digestion fragments).^4,5)^ HDX-MS is also useful for studying macro-protein complexes and for screening intermolecular interactions because of its rapid speed of detection and ability to simultaneously detect individual macro-molecular components in small amounts of mixtures.^1,3)^

HDX-MS can be measured using two ionization procedures, electrospray ionization (ESI) and matrix-assisted laser desorption/ionization (MALDI). HDX-MS with ESI, combined with liquid chromatography (LC), has been widely applied to epitope analysis and surface analysis for the detection of site-specific interactions between proteins using commercially available specialized systems.^6–8)^ Various software platforms have been developed to analyze HDX data obtained by LC-ESI-MS.^9–14)^ LC-ESI-MS systems are operated manually with minimum error and data analysis is semi-automated. In contrast, MALDI-based HDX requires no specialized instrumentation (*e.g.*, LC) and thus the measurements can be rapidly completed at low cost. Moreover, no sample is lost during LC column elution in MALDI-based HDX.^15–17)^ Furthermore, single-charged ions generated by MALDI are not hindered by multiple-charged ion peaks or unnecessary peaks such as contributions by buffer components. For example, the structural dynamics of a multi-protein complex providing more than 50 MS peaks could be analyzed using MALDI-based HDX,^18–20)^ demonstrating that MALDI-based HDX methods permit the in-depth analysis of protein dynamics with a high level of accuracy. MALDI-based HDX is considered to be another MS-based analytical procedure for examining protein dynamics because of the difference in ionization specificity and higher mass range.^15–17)^ Furthermore, the shapes of the isotopic distribution curves in HDX contain crucial information on the multiple conformations of a protein.^21–23)^ Consequently, a dedicated software program for the highly precise analysis of isotopic distribution would be expected to dramatically enhance investigations of the structural equilibrium between multiple protein conformations. There is, however, no software currently available for analyzing the protein conformation and dynamics MALDI HDX MS data except for TOF2H.^24)^ Thus, a software program for calculating isotopic distribution would pave the way for the more efficient, reliable, and rapid analysis of protein dynamics by MALDI-HDX MS.

In this paper, we report on the development of an analytical software program “Scipas DX” (“Scipas DX”: “Single-Charged Ion Peaks Analyzing System for Deuterium eXchange”) and its application to the analysis of MALDI-HDX MS protein data (http://protein.p-desi.net/scipasDX/scipasDX.html). This software automatically and quantitatively analyzes the composition of an isotopic distribution from protein sequence data and HDX spectra using least-squares regression. The program output includes average mass shifts, the average number of atoms exchanged, average deuteration ratio, abundance ratio for exchanged atoms, and their fitted spectra. Furthermore, Scipas DX also permits the high-speed processing of multi-spectra data for human adenylate kinase 1 (AK1), a model protein for structural and dynamics analyses, thus allowing the dynamics of ligand binding by AK1 to be verified.

## MATERIALS AND METHODS

### Materials

Deuterated water (D_2_O, 99.96% D : H ratio) and trifluoroacetic acid-D (99.5% D : H ratio) were purchased from Euriso-Top (Saint-Aubin, France). Pepsin, adenosine 5′-(α,β-methylene) diphosphate, and acetic acid-OD (99% D : H ratio) were obtained from Sigma-Aldrich (St. Louis, MO, USA).

### Cloning of human AK1

Total RNA (1 μg) from HEK293MSR was reverse-transcribed to template cDNA at 55°C for 60 min using the oligo(dT) anchor primer and SuperScriptIII RNase H-Reverse Transcriptase (Invitrogen, Carlsbad, CA, USA). The AK1 cDNA was produced by the polymerase chain reaction using the primers 5′-TTT TGA ATT CAT CGA AGG TCG TAT GGA AGA GAA GCT GAA GAA AAC-3′ and 5′-TTT TGT CGA CCT ACT TTA GGG CGT CCA GG-3′. The open reading frame (ORF) of AK1 was subcloned in-frame into pGex 6p-1 (GE Healthcare, Chicago, IL, USA) at *Eco*RI/*Sal*I. The cutting sequence for the PreScission Protease was inserted into the 5′ terminal of the AK1 ORF by the polymerase chain reaction using the primers 5′-AGG GGC CCA TGG AAG AGA AGC TGA AG-3′ and 5′-CTT CCA TGG GCC CCT GGA ACA GAA C-3′. Subcloned inserts were sequenced on an ABI PRISM 310 Genetic Analyzer (Applied Biosystems, Foster City, CA, USA) using a Big-Dye sequencing kit (Applied Biosystems) and pGEX sequencing primers.

### Preparation of AK1

AK1 expression vectors were transformed into BL21 (DE3) and the bacteria were incubated in LB broth with ampicillin at 37°C to an OD600 of 0.8. Protein expression was induced by the addition of 1 mmol/L isopropyl-β-thiogalactopyranoside for 4 h at 37°C. Cell pellets were resuspended in a buffer containing 20 mM Tris–HCl (pH 7.5), 100 mM NaCl, and 0.1% Tween 20, sonicated twice for 5 min at 4°C, then centrifuged at 20,000×g for 30 min. The supernatant was incubated with Glutathione Sepharose 4B (GE Healthcare) for 1 h at 4°C. The resin was then washed five times with the same buffer, and the glutathione S-transferase tag was cleared by the addition of Precision Protease (GE Healthcare) followed by further incubation for 16 h at 4°C. Glycine and proline were added to the N-terminal of AK1 to allow the digestion of the glutathione S-transferase tag by the Precision Protease. AK1 was purified by size-exclusion chromatography (Superdex 75; GE Healthcare). The AK1 protein was stored at −80°C in phosphate-buffered saline with 10% glycerol and 9.3 mM magnesium acetate. AK1 in an ADP analog binding state was prepared by adding AK1 to the analog at a ratio of 5 : 1.

### HDX reaction

Deuterium incorporation was initiated by mixing 9 μL of D_2_O with 1 μL aliquots of an approximately 50 μM AK1 solution in 0.2 mL tubes at 25°C. The H : D atomic ratio was 1 : 9. The pH of the mixture during the reaction was pH 6.8. After 1, 3, 5, 7, 10, 20, and 40 min, the exchange reactions were separately quenched by adding 3 μL of 20% acetic acid (H : D=1 : 9, pH 2.4), digested with pepsin for 3 min, then frozen with liquid nitrogen. Reactions at all time-points (1, 3, 5, 7, 10, 20, and 40 min) were prepared in independent tubes. We also examined HDX in angiotensin II incubated in D_2_O at 80°C for 15 min in order to evaluate the back-exchange and calculation accuracy of Scipas DX.

### Pepsin digestion

AK1 was digested with pepsin to obtain digestion fragments. A pepsin solution (2 μL, 1 mg/mL) was added to a mixture composed of 3 μL of 20% acetic acid and 10 μL of AK1 solution. Pepsin digestion was carried out for 3 min at pH 2.4 and 0°C.

### MALDI mass spectrometry

Quenched samples from each of four data points were mixed with 20 μL of 10 mg/mL α-cyano-4-hydroxycinnamic acid 50% acetonitrile containing 0.025% triﬂuoroacetic acid (H : D=1 : 9), then loaded on a precooled sample plate at 10°C in dry air. The matrix solution was precooled on ice for 5 min. At 30 s after loading the sample plate in the MALDI-time of flight (TOF) mass spectrometer at a pressure of 7 Pa, the pressure was decreased to 10^−5^ Pa. HDX MS spectra were obtained using an Ultraflex III, MALDI TOF/TOF instrument (Bruker Daltonics Inc., Billerica, MA, USA). The *m*/*z* values were externally calibrated by peptide II and protein I standards (Bruker Daltonics Inc.). Peptides whose isotope masses were not clearly separated were measured in the linear mode and were used only for HDX-kinetic analysis with their average masses. Peptic fragments were identified by accurate mass, MS/MS analysis, and mutation. The percent deuterium content of fragments and HDX kinetics were calculated from average masses using graphing software (ORIGINPRO 8J; OriginLab, Northampton, MA, USA) as described previously.^25)^ The HDX time course was analyzed using the following equation: 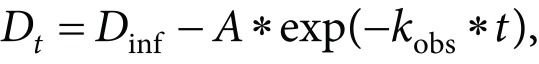
(1) where *D_t_* and *D*_inf_ are the fractions of deuterium incorporation at exchange times *t* and infinity, respectively; *A* is the fraction of exchangeable protons that can be detected during the exchange time; and *k*_obs_ is the apparent first-order rate constant of the HDX reaction.^26)^ The observed *k*_obs_ value represents the average of all of the exchange rates of the different amide protons. In this study, reactions at all time-points were independently prepared in different tubes (*n*>3) and error bars in Fig. 4 represent the SD of these samples.

## RESULTS AND DISCUSSION

### Development of the Scipas DX software program

**Figure 1:**
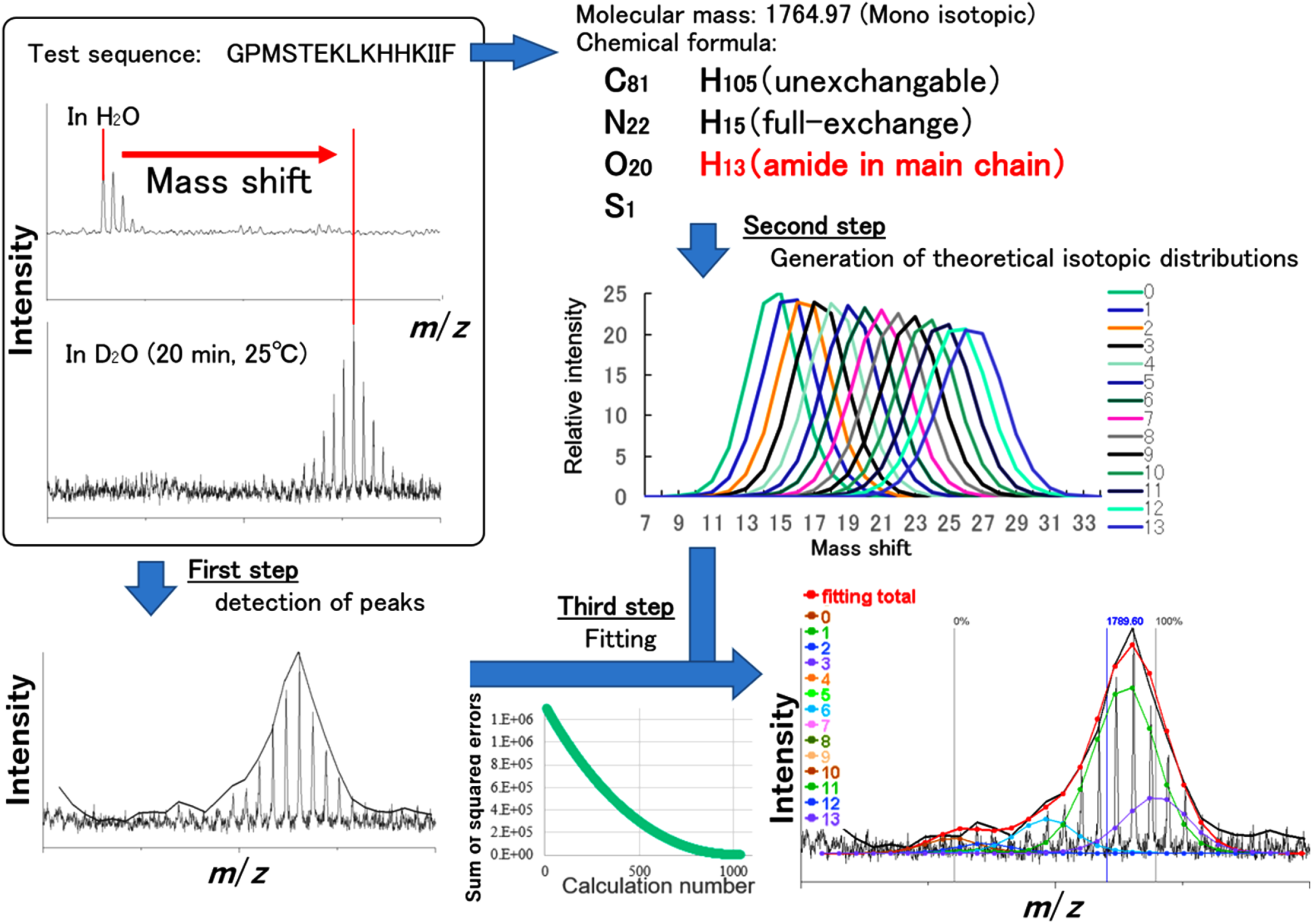
Fig. 1. HDX analysis by Scipas DX.

We developed the original software program Scipas DX to analyze the level of exchange of deuterium using a mass spectra for the HDX measurements. An HDX reaction sample is composed of protein fragments with various numbers of deuterium atoms incorporated. Scipas DX automatically and quantitatively analyzes the composition of the isotopic distribution using sequence data and HDX spectra by least-squares regression. This software program was written in Java and focuses only on single-charged ion peaks generated by MALDI (exchange conditions for H : D=1 : 9). The input format of the spectrum data supports ASCII, and Scipas DX can be applied to data from a variety of vendors. Scipas DX has four graphical user interfaces: spectra, sequence, HDX, and exchange number windows (Fig. S1). Protein sequence data are read into the sequence window. Protein fragments identified by accurate mass, MS/MS measurements, and mutations are listed in an HDX management table in the HDX window by the operator. The exchange ratios of all target peptides are automatically calculated using observational data. Figure 1 shows the three steps of the Scipas DX-directed computation process for a virtual peptide fragment (GPMSTEKLKHHKIIF): (1) detection of peaks, (2) generation of theoretical isotopic distributions from the target sequence, and (3) fitting of the observational data, which enables the automatic calculation of the HDX ratios of all target protein fragments. In the first step, peaks for all detectable isotopic masses of a test peptide in an HDX experiment are converted into quantitative values using the peak area (the *default* mass range is the theoretical mass ±0.3 Da). In the second step, Scipas DX generates a set of all isotopic mass distributions for the number of deuterium atoms incorporated with reference to the chemical formulas of the target fragments.^27)^ In the third step, theoretical isotopic distributions are fitted to the observed curve by least-squares regression. Collectively, these computational processes indicate that Scipas DX outputs the average number of exchanged atoms, the average deuteration ratio, the abundance ratio for exchanged atoms, and their fitted spectra for the virtual GPMSTEKLKHHKIIF peptide (Fig. 1). Information regarding side chains, which are completely exchanged during the acid quenching, is computationally removed under exchange condition of H : D=1 : 9, as previously reported.^1,3)^ The test calculation for a virtual spectrum demonstrated that the Scipas DX-analyzed abundance ratios are in good agreement with the theoretical ones (Fig. S2), confirming the validity of the Scipas DX calculations for abundance ratios for all components.

This software converts all detectable isotopic masses of target peptides to quantitative values from the peak area in the observed spectrum. In addition, all isotopic mass distributions of different numbers of incorporated deuterium atoms are generated from the chemical formula of the target peptide (sequence example: GPMSTEKLKHHKIIF). Scipas DX fits theoretical isotopic distributions to the quantitative converted peak by least-squares regression and calculates the average mass and number of deuterium-exchanged peptide peaks.

We subsequently examined the back-exchange reaction of MALDI HDX and the calculation accuracy of Scipas DX for the experimental data using angiotensin II. All six amide hydrogen atoms in angiotensin II were deuterated during incubation in D_2_O at 80°C for 15 min (Fig. S3). the Scipas DX analysis demonstrated that a total of 98.95% of the hydrogen atoms in the angiotensin II sample had been replaced with deuterium atoms (5.937 deuterium atoms distributed between the 6 sites). The analysis showed that all six hydrogen atoms were exchanged with deuterium in 93.7% of the angiotensin II molecules and five hydrogen atoms were substituted with deuterium in 6.3% of the angiotensin II molecules. These data indicate that Scipas DX precisely detected the HDX of angiotensin II with an experimental error of 1.05% including back exchange, thus confirming the high accuracy of Scipas DX. Altogether, these results verified that the Scipas DX analysis of the present MALDI-HDX MS data was performed with markedly low back exchange and high accuracy. The respective peptide fragments are presented as a composition profile of abundance ratios for exchanged atoms in the exchange number window (Fig. S3). The isotopic distribution of a target fragment can be enlarged in the spectral window by selecting that fragment in the HDX table, and all components fitted by the calculation are displayed on the observed spectra (Fig. S3). It is noteworthy that Scipas DX enables the automatic processing of all spectra in the same folder in one operation. Altogether, these results substantiate the utility of Scipas DX in an HDX analysis based on observed mass data for peptide fragments.

### Calculation of HDX in AK1 using Scipas DX

We subsequently analyzed HDX and the dynamics of the model protein AK1 using Scipas DX. AK1 is a highly conserved enzyme that is involved in energy metabolism and catalyzes the phosphoryl exchange reaction ATP+AMP↔2ADP.^28,29)^ AK1 has been extensively investigated as a model protein in structural biology using X-ray crystallography, nuclear magnetic resonance (NMR), and molecular dynamics simulation.^30–32)^ Both the closed and opened structures of ligand-binding pocket of AK1 were observed.^30–32)^ AK1 was found to adopt the opened structure mainly in the apo state, and the closed structure to form a reaction site for the phosphate transfer of ATP to AMP by binding them.^30–32)^ The reacted products, ADP, are then released when the ligand pockets open.^30–32)^ The release of ADP from the ligand-binding pocket was shown to be the rate-limiting step and to cause structural changes in AK1.^30)^ We also analyzed the ligand-dependent structural change in human AK1 by MALDI-TOF MS-based HDX using Scipas DX.

**Figure 2:**
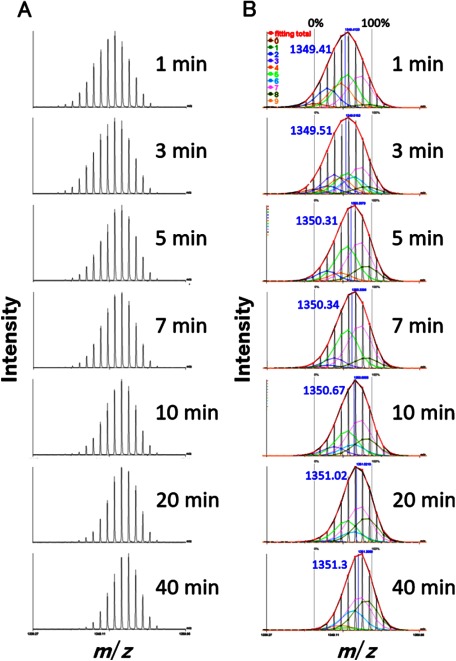
Fig. 2. Time–course analysis of HDX mass spectra of the 107–117 region (sequence: FERRIGQPTLL) in AK1.

**Figure 3:**
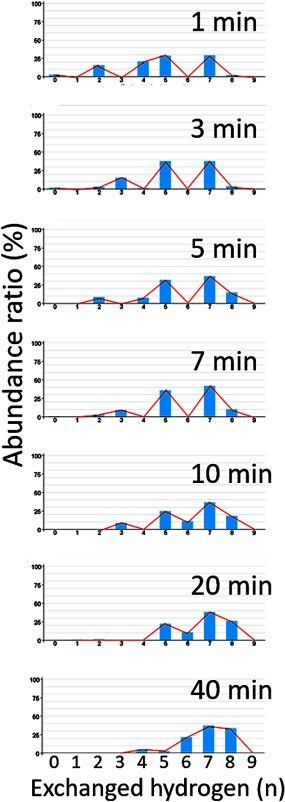
Fig. 3. Time-dependent composition profiles of the 107–117 region (sequence: FERRIGQPTLL) in AK1.

AK1 was incubated in 90% D_2_O in the presence (closed structure) or absence (opened structure) of an ADP analog, adenosine 5′-(α,β-methylene) diphosphate, followed by pepsin digestion (Fig. S4). We comprehensively covered the AK1 sequence by selecting eight regions of analysis: residues 1–14, 14–45 (the HDX data between 1–45 and 1–14 was subtracted), 46–75, 83–106, 107–117, 119–131, 131–165 (the HDX data between 119–165 and 119–131 were subtracted) and 166–196 (Fig. S5). The HDX spectra of these protein fragments at seven time points were automatically analyzed using Scipas DX and the average deuteration ratio (Fig. 2) and abundance ratio for the exchanged atoms were calculated (Fig. 3) and used for subsequent kinetics analyses and to detect the existence of a slow equilibration of local structures, respectively. It is noteworthy that several days were required for the manual high-precision HDX analysis of AK1 (including test experiments) without Scipas DX, whereas using Scipas DX, it was possible to automatically complete all calculations in only a few minutes. These results confirm both the precision and speed of these MALDI MS-based HDX analyses.

(A) Time–course analysis of HDX mass spectra of the 107–117 region without Scipas DX. (B) Scipas DX-directed time–cource analysis of mass spectra of the 107–117 region. Red lines show the summation of deuterium atoms incorporated (shown in other colors). Blue numbers are the average masses.

Composition profiles for the number of incorporated deuterium atoms into the 107–117 fragment. Blue bars show the abundance ratio for exchanged atoms and red lines connects the tops of their bars.

Most of the fragment peaks of AK1 showed wide isotopic distributions after 1 min of the HDX reaction, and their distributions were shifted to higher masses and became sharper over time (Fig. 2A). The HDX-MS spectra were obtained as a single isotopic distribution without Scipas DX (Fig. 2A). Consequently, based on the peak pattern, the single distribution of fragment 107–117 appeared to shift as the number of atoms exchanged increased during the HDX process (Fig. 2A). The use of Scipas DX resulted in a high accuracy (red lines in Fig. 2B), however, but the existence of diverse isotopic distributions containing different numbers of exchanged deuterium atoms was observed (in other colors in Fig. 2B). In addition, the use of Scipas DX permits the composition profiles to be quantitatively output, and indicates the presence of diverse numbers of incorporated deuterium atoms within several minutes after the initiation of the HDX reaction (Fig. 3). These composition profiles for deuterium exchange and the observed wide isotopic distributions show that multiple conformations that are in slow equilibrium are involved in the stability of the active protein and these conformations function as a functional switch between the active and inactive form of the protein.^21–23,33)^ For example, the profile of fragment 107–117 after a 1 min incubation was 2.8, 0, 16.0, 0, 20.8, 28.7, 0, 29.3, 2.3, and 0.0% for 0 to 9 deuterium incorporations (blue bars in Fig. 3), confirming that of three populations are generated (red lines in Fig. 3). After a 40 min incubation, a nearly single distribution of the numbers of incorporated deuterium atoms was observed: 0.1, 0, 0, 0, 5.0, 2.5, 21.6, 37.1, 33.7, and 0% for the number of incorporated deuterium atoms (red lines in Fig. 3). These results provide evidence that the 107–117 region adopts multiple conformations in slow equilibrium, during which multiple hydrogens are exchanged with deuterium at the same time. Other peaks also showed multiple distributions of the numbers of incorporated deuteriums, such as fragment 83–106 (Fig. S6). Collectively, these data (using MALDI-HDX) show the first simultaneous detection of multiple conformations of AK1 in slow equilibrium.

### Kinetics analysis of HDX in AK1

**Figure 4:**
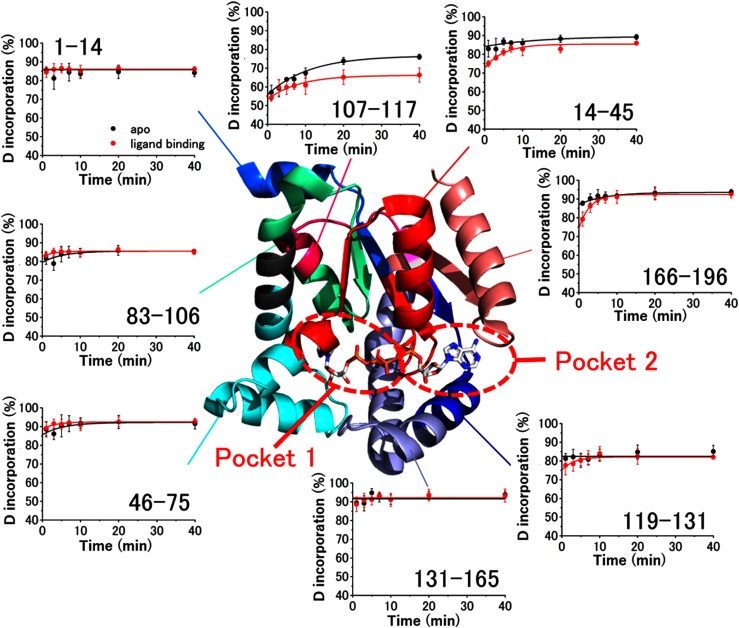
Fig. 4. Analyzed peptide fragments in AK1 and their HDX time courses.

We analyzed the kinetics of HDX by fitting the shifts in the individual average masses of the aforementioned eight fragments as a function of time using Eq. (1) to calculate the HDX parameters *D*_0_, *D*_inf_, *A*, and *k* (Fig. 4 and Table 1). The rate constant, *k*, for most of the fragments increased upon binding to the ADP analog. The fractions of deuterium incorporated, *D*_0_ and *D*_inf_, mainly reflect the accessibility of solvent and structural flexibility, respectively, which serve to protect hydrogen atoms from the solvent.

Analyzed fragments are shown on the AK1 X-ray structure with different colors (PDB: 1Z83): blue, red, light blue, green, pink, deep blue, purple, and salmon pink show residues 1–14, 14–45, 46–75, 83–106, 107–117, 119–131, 131–165, and 166–196, respectively. The red broken circles show two ligand-binding pockets. HDX time courses of the apo form and the ADP analog binding state are plotted as black and red dots, respectively, and their fitting curves were calculated with Eq. (1).

**Table 1:** Table 1. HDX kinetic parameters of the apo and ADP analog binding states of AK1.

apo					
No.	Residues	*D*_inf_	*A*	*k*_obs_	*D*_0_
1	1–14	85.8±0.3	0.0±0.0	—±—	85.8
2	14–45	89.6±1.2	5.5±1.2	0.07±0.06	84.1
3	46–75	92.2±1.2	6.6±2.6	0.18±0.15	85.6
4	83–106	85.5±0.6	6.1±2.3	0.19±0.12	79.4
5	107–117	76.7±0.9	20.8±1.5	0.12±0.03	55.8
6	119–131	82.4±0.7	0.0±0.0	—±—	82.4
7	131–165	91.6±0.7	0.0±0.0	—±—	91.6
8	166–196	92.2±0.8	8.8±18.6	0.02±0.06	83.3
ADP analog binding					
No.	Residues	*D*_inf_	*A*	*k*_obs_	*D*_0_
1	1–14	86.1±0.2	0.0±0.0	—±—	86.1
2	14–45	85.4±0.7	12.8±2.0	0.20±0.06	72.5
3	46–75	92.5±0.4	4.0±1.6	0.31±0.16	88.5
4	83–106	85.5±0.4	2.9±1.6	0.34±0.26	82.6
5	107–117	66.2±1.3	13.0±1.5	0.12±0.03	53.2
6	119–131	82.1±0.3	7.2±3.0	0.32±0.16	75.0
7	131–165	88.5±1.4	0.0±0.0	—±—	88.5
8	166–196	88.3±1.0	20.9±4.2	0.42±0.14	67.4

HDX kinetic parameters were obtained by fitting the time courses for each fragment with Eq. (1). *D*_inf_ and *D*_0_ were mainly determined by the exchangeable hydrogen atom ratio and the degree of solvent exposure, respectively. The rate constant *k*_obs_ shows the equilibrium between exchangeable and nonexchangeable hydrogen atoms in the local structure, and *A* is the hydrogen ratio in the equilibrium state.

Two regions of AK1, residues 46–75 and 131–165, play important roles in the binding of two substrate molecules and the conformations of these regions are dramatically altered upon ligand binding^30,31,34)^ as shown by X-ray crystal structures, NMR, and molecular dynamics analyses.^31)^ These findings suggest that regions 46–75 and 131–165 adopt flexible structures in order to release the products. The present HDX analysis shows that approximately 90% of the hydrogen atoms in these regions undergo exchange with deuterium within a few minutes (Fig. 4), indicating the structural flexibility of these regions. Thus, the present HDX analysis data are compatible with previously reported data obtained by an X-ray crystal structural, NMR, and molecular dynamics analyses.

We subsequently analyzed the dynamics of AK1 in the presence of its specific ligand. The resulting fitting curves revealed that the ligand binding-induced HDX parameter changes in three regions: residues 14–45, 107–117, and 166–196. The HDX of amide hydrogen atoms was inhibited by binding to ADP analogs in fragment 166–196 at *t*=0 and at infinity in fragment 107–117. Deuterium incorporation in fragment 14–45 was decreased by analog binding over the entire time range that was investigated. The ADP analog binds to two pockets in AK1 (pockets 1 and 2 in Fig. 4), and this results in a structural change from the opened to the closed form. An X-ray structure analysis previously showed that residues 14–45 form pockets 1 and 2 (PDB: 1Z83). The incorporation of fragment 14–45 at the ligand binding state (*D*_0_=72.5 and *D*_inf_=85.4±0.7) was clearly lower than at the apo state (*D*_0_=84.1 and *D*_inf_=89.6±1.2), as shown in Fig. 4 and Table 1. This decrease in deuterium incorporation indicates a reduced solvent accessibility to the ligand-binding pockets due to the formation of a stable closed structure. In contrast, the region comprising residues 166–196 is involved in forming part of pocket 2 and shows a different tendency for *D*_inf_, namely, *D*_0_ for the ligand binding state (*D*_0_=67.4) was 20% lower than for the apo state (*D*_0_=83.3) but increased and was similar to that of the apo state after a 10 min incubation (Fig. 4). These results serve to demonstrate that a ligand in pocket 2 inhibits HDX in the 166–196 region at time 0 and that the frequent conformation change due to the release of the temporal ligand from the pocket slowly facilitates deuterium incorporation into this region. Consequently, we conclude that this region plays a newly identified key role in controlling the ligand affinity of pocket 2 and product release, compatible with the finding that residues 166–196 decrease both the substrate affinity and the catalytic efficiency.^35)^ Collectively, the consistency of the present MALDI-HDX results with previously reported steady-state kinetics analyses of the AK1 enzyme reaction^36)^ and new relevant findings (Fig. 4) confirm the accuracy, reliability, and versatility of Scipas DX-assisted MALDI HDX in the analysis of protein dynamics.

We detected another novel dynamics feature of AK1. The X-ray crystal structure (PDB: 1Z83) shows that fragment 107–117 is located at some distance from the ligand binding pockets but the HDX parameter *D*_inf_ of apo-AK1 (*D*_inf_=76.7±0.9) was unexpectedly affected by the binding of the ADP analog (*D*_inf_=66.2±1.3) (Fig. 4 and Table 1). Since a low *D*_inf_ is generally indicative of poor structural flexibility of a protein,^21,25,33)^ these results show that residues 107–117 indirectly lose their flexibility as the result of ligand binding to pocket 1. Taken together, the present HDX analyses verify that the molecular mechanism underlying ligand binding to pocket 1 is distinct from that of pocket 2. This is the first report of a differential mode of ligand binding at the two binding sites in AK1. The present rapid and accurate MALDI-HDX analysis using Scipas DX verified that the 107–117 region is one of the most important regions for ligand binding, as shown by both the average mass change and the profiles of the exchanged atoms.

In conclusion, we report on the development and evaluation of an analytical software program “Scipas DX” for use in the MALDI MS-based HDX analysis of proteins. This software automatically and quantitatively analyzes the composition of an isotopic distribution from sequence data and HDX spectra by least-squares regression within a few minutes. MALDI MS-based HDX of AK1 using Scipas DX revealed i) differences in the dynamics of two substrate pockets; ii) the regions encompassing residues 107–117 and 166–196 are novel candidates for controlling the activity of AK1; and iii) the existence of a slow equilibration between multiple conformations. These results shed light on the usefulness and versatility of using a combination of Scipas DX and MALDI-HDX MS in investigating a wide variety of dynamics involved in molecular recognition and interactions.

## Abbreviations

AK1, adenylate kinase 1; ESI, electrospray ionization; HDX, hydrogen/deuterium exchange; LC, liquid chromatography; MALDI, matrix-assisted laser desorption/ionization; MS, mass spectrometry; ORF, open reading frame; TOF, time of flight

## Notes

Mass Spectrom (Tokyo) 2019; 8(2): S0082
