# Supplementary material for: Development of Software for the In-Depth Analysis of Protein Dynamics as Determined by MALDI Mass Spectrometry-Based Hydrogen/Deuterium Exchange
Source: Mass Spectrom (Tokyo). 2020 Feb 14;8(2):S0082. doi: 10.5702/massspectrometry.S0082 (PMC7709884; doi:10.5702/massspectrometry.S0082)

Development of software for deep analysis of protein dynamics as determined by MALDI mass spectrometry-based hydrogen/deuterium exchange

Tatsuya Yamamoto, Tohru Yamagaki, and Honoo Satake

Bioorganic Research Institute, Suntory Foundation for Life Sciences, Kyoto 619-0284, Japan

*Corresponding author: Honoo Satake,
Bioorganic Research Institute, Suntory Foundation for Life Sciences, Kyoto 619-0284, Japan
E-mail address: satake@sunbor.or.jp

Supporting information

Fig. S1 Overview of Scipas DX software. The graphical user interface is comprised of four windows: Spectra, Sequence, HDX, and Exchange number.

Fig. S2 Test calculation of the Scipas DX using a virtual spectrum. The test calculation was carried out using the virtual spectrum that sums the isotopic distributions with a theoretical abundance ratio (from 0.2 to 1.4) for exchanged atoms. Scipas DX accurately calculated the abundance ratios of all components in this test.

Fig. S3 100% HDX test for angiotensin II. We tested the HDX of angiotensin II at 80°C for 15min and found that 98.95% of the hydrogen atoms exchanged with deuterium atoms (5.937 deuterium atoms distributed between the 6 sites). The analysis showed that all six hydrogen atoms were exchanged with deuterium in 93.7% of the angiotensin II molecules and five hydrogen atoms were substituted with deuterium in 6.3% of the angiotensin II molecules. These data indicate that Scipas DX precisely detected the experimental error as 1.05% including back exchange, thus confirming the high accuracy of Scipas DX.

Fig. S4 MALDI mass spectrum of pepsin-digested AK1 in HDX (the exchange time was 1min). Each of the AK1 pepsin-digests is assigned to the corresponding mass value.

Fig. S5 HDX-analyzed fragments in the AK1 sequence. Gray and white bars show the

primary structure of AK1 digested by pepsin, and bars colored in gray are the directly detected regions. Asterisks (*) indicate fragments measured in the linear mode by MALDI TOF MS. Data analysis was carried out on eight regions: 1–14, 14–45 (HDX data between 1–45 and 1–14 were subtracted), 46–75, 83–106, 107–117, 119–131, 131–165 (HDX data between 119–165 and 119–131 were subtracted), and 166–196.

Fig. S6 The HDX spectra and composition profiles of peptic fragment 83–106 (sequence: VAKVNTSKGFLIDGYPREVQQGEE) in AK1 with time. (A) Analysis of the mass spectra of fragment 83–106, as shown in different colors. Red lines are the summation of the different incorporation numbers represented by other colors. Blue numbers show the average masses. (B) Composition pro-les for the number of incorporated deuterium atoms into fragment 83–106. Blue bars show the abundance ratio for exchanged atoms and red lines connect tops of their bars.

Fig. S1 Overview of Scipas DX software.

Overview

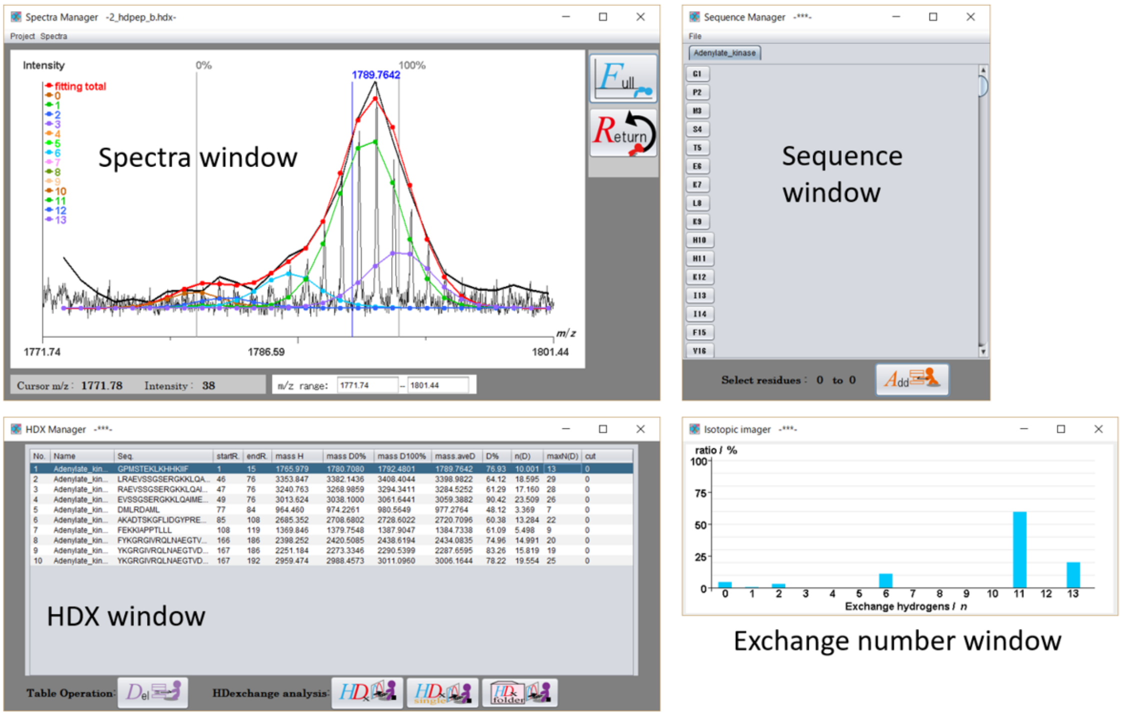

Fig. S2 Test calculation of the Scipas DX using a virtual spectrum.

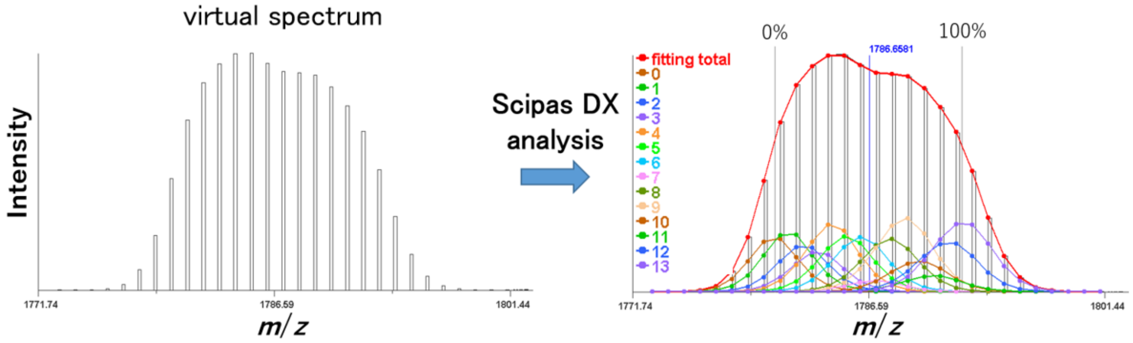

Exchange number	0	1	2	3	4	5	6	7	8	9	10	11	12	13
**Theoretical abundance**	0.9	1.0	0.8	0.7	1.2	1.0	1.0	0.2	1.0	1.4	0.6	0.3	1.0	1.4
**Calculated abundance**	0.9	1.0	0.8	0.7	1.2	1.0	1.0	0.2	1.0	1.4	0.6	0.3	1.0	1.4

Fig. S3 100% HDX test for angiotensin II.

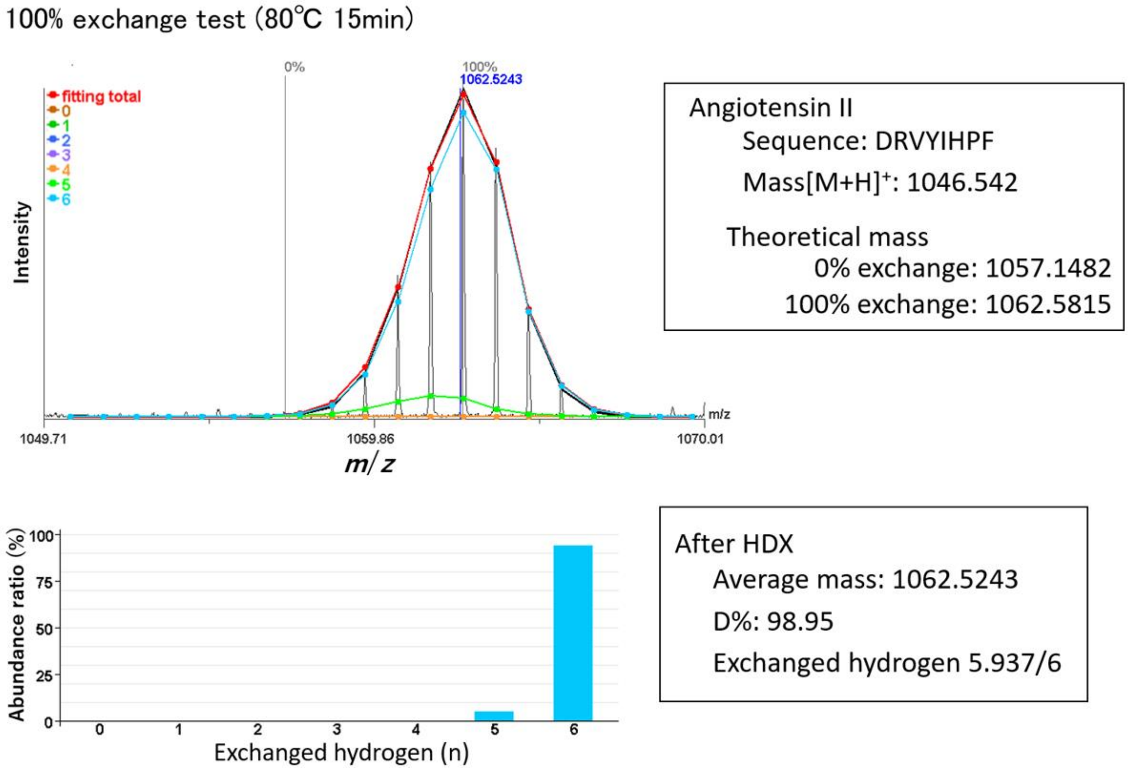

Fig. S4 MALDI mass spectrum of pepsin-digested AK1 in HDX (the exchange time was 1min).

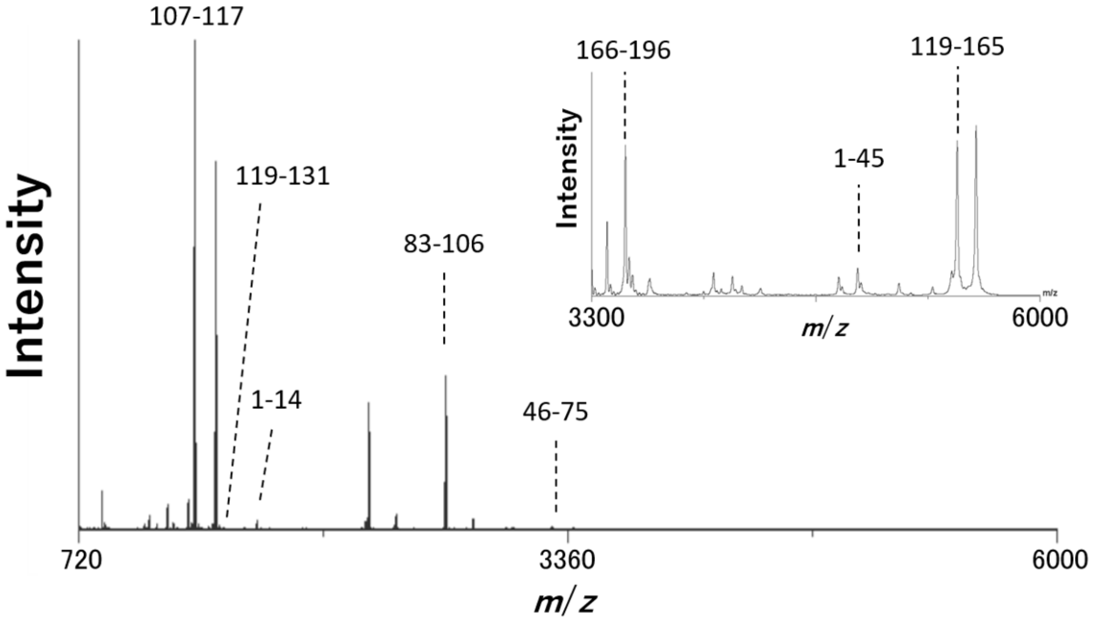

Fig. S5 HDX-analyzed fragments in the AK1 sequence.

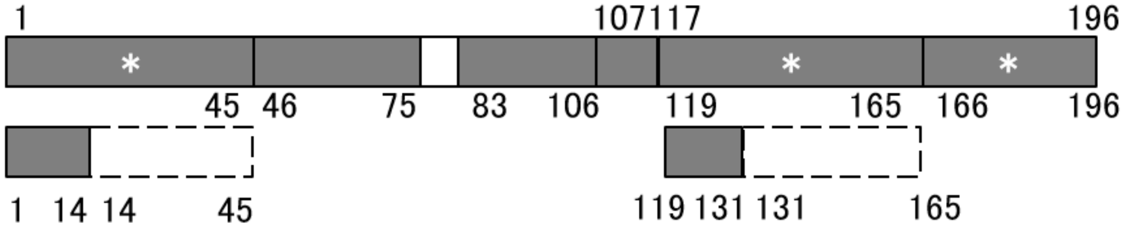

Fig. S6 The HDX spectra and composition profiles of peptic fragment 83–106 (sequence: VAKVNTSKGFLIDGYPREVQQGEE) in AK1 with time.

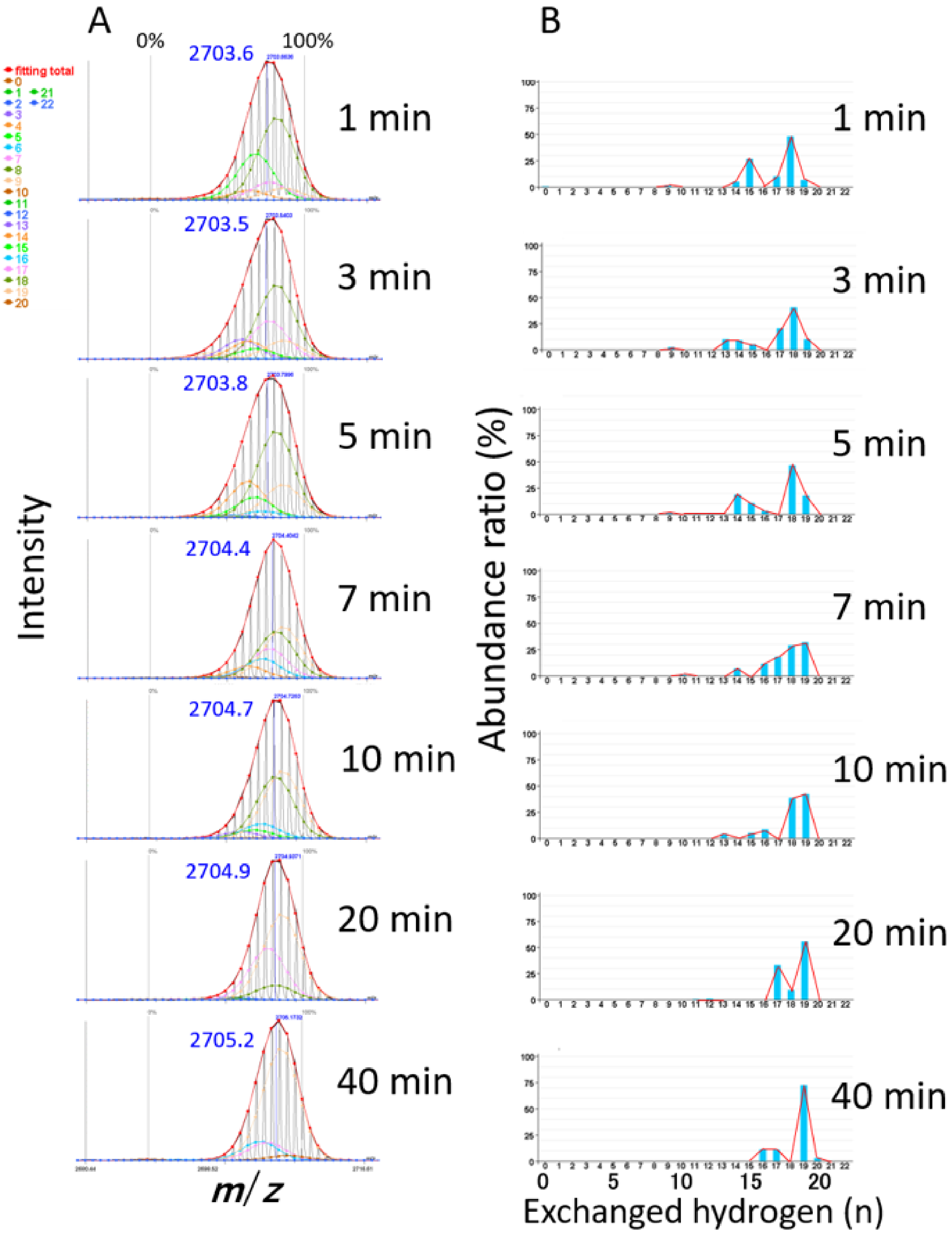

Supplement: Supplementary Data [file massspectrometry-8-2-S0082-s001.pdf]
